# Functions and clinical applications of exosomes in pancreatic cancer

**DOI:** 10.1007/s11033-022-07765-8

**Published:** 2022-09-12

**Authors:** Zhichen Jiang, Huiju Wang, Yiping Mou, Li Li, Weiwei Jin

**Affiliations:** 1 Department of General Surgery，Devision of Gastroenterology and Pancreas, Zhejiang Provincial People’s Hospital, Affiliated People’s Hospital, Hangzhou Medical College, 310014 Hangzhou, Zhejiang China; 2 The Second School of Clinical Medicine, Zhejiang Chinese Medical University, 310053 Hangzhou, Zhejiang China; 3 Key Laboratory of Gastroenterology of Zhejiang Province, 310014 Hangzhou, Zhejiang China; 4 Cancer Center, Zhejiang Provincial People’s Hospital, Affiliated People’s Hospital, Hangzhou Medical College, 310014 Hangzhou, Zhejiang China

**Keywords:** Exosome, Extracellular vesicles, Pancreatic cancer, Biomarker, Therapy

## Abstract

Pancreatic cancer (PC) is one of the most malignant tumors and has an abysmal prognosis, with a 5-year survival rate of only 11%. At present, the main clinical dilemmas in PC are the lack of biomarkers and the unsatisfactory therapeutic effects. The treatments for and outcomes of PC have improved, but remain unsatisfactory. Exosomes are nanosized extracellular vesicles, and an increasing number of studies have found that exosomes play an essential role in tumor pathology. In this review, we describe the process of exosome biogenesis, as well as exosome extraction methods and identification strategies, and we then explain in detail the roles and mechanisms of exosomes in invasion, metastasis, chemoresistance and immunosuppression in PC. Finally, we summarize the clinical applications of exosomes. Our observations indicate that exosomes represent a novel direction in the clinical treatment of PC.

**Supplementary Information:**

The online version contains supplementary material available at 10.1007/s11033-022-07765-8.

## Introduction

PC is a common malignant tumor in the digestive system, that is stealthy, highly invasive, rapidly progressive, and deadly. It has the lowest 5-year survival rate of all tumors, at 11%[1]. According to data released by the American Cancer Society, PC is estimated to affect more than 62,210 individuals and lead to over 49,830 deaths in the United States in 2022[1].In recent years, the incidence of PC has increased significantly. By 2030, the total number of deaths from PC will increase dramatically, and PC will become the second leading cause of cancer-related death[2].

The current clinical outcomes for PC are poor. The leading causes of the poor prognosis of PC are the lack of early symptoms, late clinical diagnosis, early metastasis, and resistance to chemo/radiotherapy[1, 3, 4]. Surgical excision plus systemic adjuvant chemotherapy is the preferred treatment for PC. Among these strategies, surgery is the only possible solution for the radical cure of PC. However, less than 20% of patients are candidates for surgery because most patients already have advanced-stage disease at diagnosis[2]. With the continuous development of medical technology and in-depth research on tumor pathology and mechanisms, the treatment of PC has been further optimized. Neoadjuvant therapy[5], targeted therapy[5, 6], immunotherapy[7], and improved chemotherapy[4] are emerging options in the treatment of PC. These approaches allow a subset of patients with vascular invasion or advanced-stage to have a chance for surgery[8] and further extension of survival. However, these treatments cannot achieve ideal results, and new treatments for PC are still urgently needed.

Exosomes are extracellular vesicles that are secreted by cells and have a diameter in the range of 40 to 150 nm [9]. In recent years, studies have found that exosomes can promote invasion, metastasis, chemoresistance, immunosuppression, and other functions in tumors and show great potential value for diagnosis, prognostic assessment, treatment, and other clinical applications in cancers[10]. In this review, we summarized and analysed the role of exosomes in invasion, metastasis, chemoresistance, and immunosuppression in PC, and aimed to off new ideas for developing a novel treatment for PC.

## Characteristics, extraction, and identification of exosomes

### Characteristics of exosomes

**Fig. 1:**
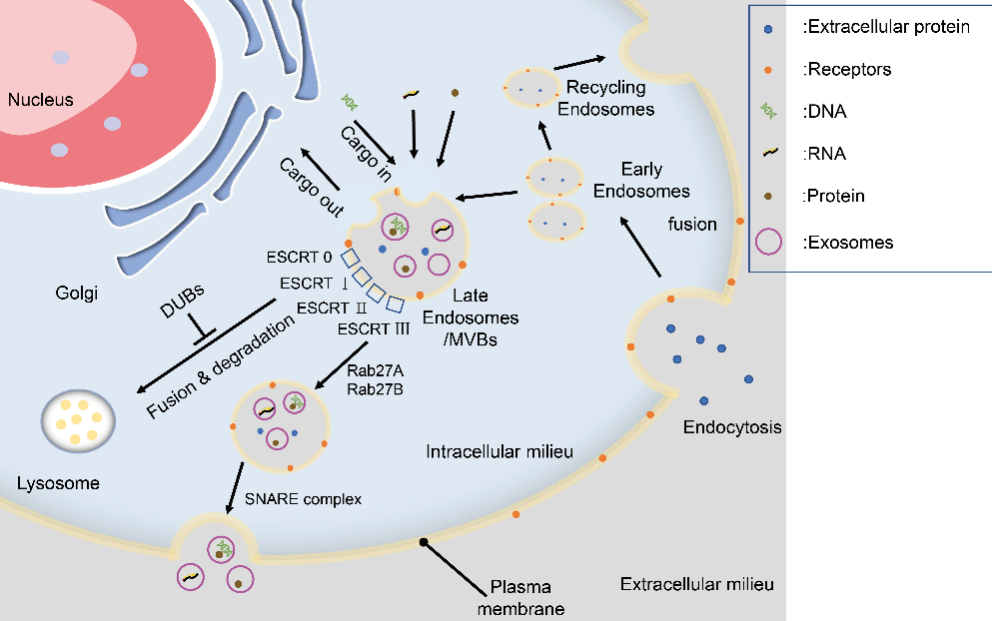
The biogenesis of exosomes

Exosomes are extracellular vesicles that can be extracted from various body fluids (including blood, lymph, urine, cerebrospinal fluid, saliva, etc.) and are 40–150 nm in diameter[9]. The discovery of exosomes in reticulocytes by Pan and Johnstone in the 1980s brought exosomes to attention for the first time[11]. Currently, the mainstream view still holds that exosomes are extracellular vesicles secreted by parental cells through endogenous pathways[10, 12] (Fig. 1). The process of exosome biogenesis can be roughly divided into several stages[13–15]. First, the cell membrane invaginates to form primary endocytic vesicles, which fuse to form early endosomes (EEs). Subsequently, the EEs return to the plasma membrane as recycled endosomes for release or conversion to late endosomes(LEs), also called multivesicular bodies (MVBs), Subsequently, through regulation by endosomal-sorting complex required for transport (ESCRT) family members, MVBs bud inward to form intraluminal vesicles (ILVs) and are then, through regulation by Rab27 subfamily proteins, such as Rab27A and Rab27B, induced to translocate to the periphery of the cell, Finally, with the assistance of the sensitive factor attachment protein receptor (SNARE) complex, MVBs fuse with the plasma membrane and release ILVs - namely, exosomes - into the extracellular space. Therefore, exosomes have the same bilayer lipid membrane structure as the cell membrane[9, 12] and contain membrane- and cytoplasm-derived substances, such as proteins, RNA, DNA, lipids, and metabolites, from the parental cell[16]. Initially, exosomes were thought to constitute a way for cells to secrete and metabolize waste, but an increasing number of studies have found that exosomes are key mediators of physiological functions such as information exchange between cells, material transfer and antigen presentation, bringing increasing attention to exosomes[17, 18].

### Extraction of exosomes

Exosomes are nanoscale extracellular vesicles secreted by cells[19]. At present, two main problems limit basic and applied research on exosomes: (1) The lack of approaches to simplify the steps of exosome extraction and improve the extraction efficiency, and (2) The lack of ways to distinguish exosomes from other extracellular vesicles[20]. Currently, exosome extraction methods mainly fall into specific categories[12, 20–22] (Supplemental Table 1). The first category contains methods based on the difference in the density of the vesicles to be measured, such as ultracentrifugation and density gradient centrifugation. These are the most common kinds of methods, among which density gradient centrifugation is considered the gold standard for exosome insolation. Although this method is simple to operate, it has the disadvantages of producing a low yield, consuming a considerable amount of time and possibly causing damage to exosomes. The second category contains methods based on the difference in the particle size of the vesicles to be measured. The specific techniques include sequential filtration, ultrafiltration, size exclusion chromatography (SEC), etc. These methods are becoming increasingly popular among researchers because of their advantages of efficiency and ease of implementation, but they have the disadvantages of damaging exosomes and clogging the membrane with vesicles. The third category contains methods that produce antigen-antibody reactions based on the surface antigens on the vesicles; these methods have the most important advantage of high specificity but have the disadvantages of a high reagent cost and a relatively small sample size. The fourth category contains methods based on a precipitation reaction, which have the advantage of being simple to perform but are affected by low purity and specificity. The fifth category is microfluidic technology. This is an emerging exosome extraction technology that utilizes the size, density, surface antigens, and other characteristics of exosomes for extraction. This technology has many advantages, such as low sample and reagent consumption, high resolution and sensitivity, and short determination time. However, due to the great technical difficulty of these methods, further research is required[23, 24]. At present, exosome extraction technology has developed considerably, and the extraction efficiency has improved dramatically, providing conditions favourable for the study of exosomes in tumor invasion, metastasis, chemoresistance, immunosuppression, and exosome-based therapy.

#### Supplemental table 1

Extraction methods and characteristics of exosomes.

### Identification of exosomes

As exosomes are a type of extracellular vesicle, it is most important to distinguish them from other extracellular vesicles[25]. According to the particle size, source and surface molecular markers, extracellular vesicles can be classified into exosomes, microvesicles [26, 27], apoptotic bodies, and migrasomes [28, 29] (Supplemental Table 2). Currently, exosome identification mainly includes the following three aspects: 1. Particle size detection of exosome is in the range of 40–150 nm [9],but the final results of different detection methods are inconsistent and the size measurements via transmission electron microscopy (TEM) are usually smaller than nanoparticle tracking analysis (NTA) 0.2. Morphological observation through TEM is used to determine whether the vesicles have a double-layer membrane structure[9, 12]. 3. Detection of molecular markers on exosomes surfaces[30] through immunofluorescence, Western blotting (WB), flow cytometry, and other techniques is used to detect molecular markers, such as CD81, CD63, and CD9, on exosome surfaces[31–33]. A vesicle can be identified as an exosome if it meets all three criteria.

#### Supplemental table 2

Types of extracellular vesicles.

## Effect of exosomes on invasion, metastasis, chemoresistance, and immunosuppression in PC

It has been shown that tumor cells can secrete a large number of exosomes[34, 35] and promote the occurrence and development of tumors through exosomes[36–38]. In PC, exosomes play a role in regulating invasion, metastasis, chemoresistance, and immunosuppression through their contents or surface proteins.

### Exosomes are involved in invasion and metastasis in PC

**Fig. 2:**
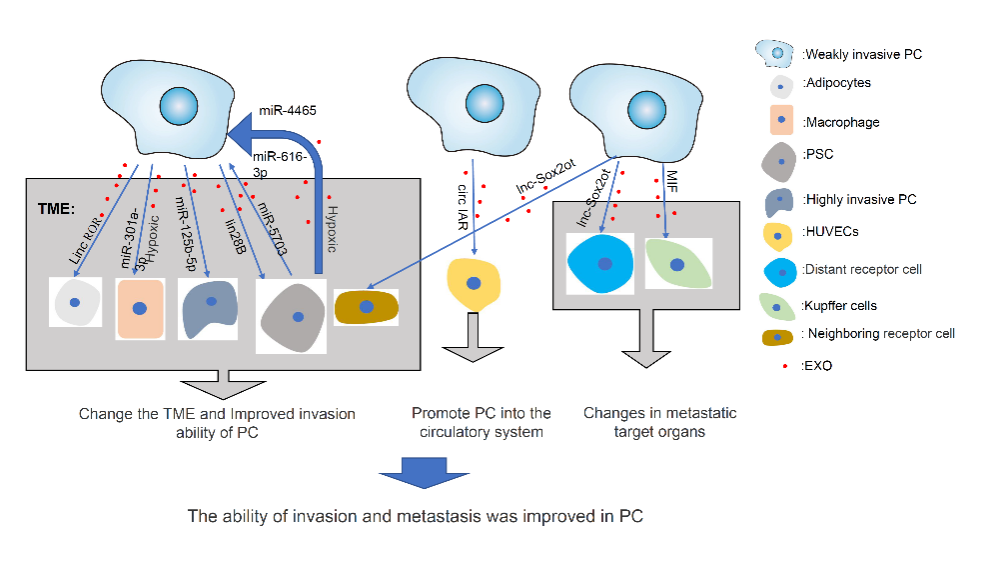
Exosomes participate in multiple processes of invasion and metastasis in PC

Invasion and metastasis are critical malignant behaviours of tumors, especially in PC[4]. Tumor invasion and metastasis are continuous and progressive processes[39, 40] that include the following specific steps: (1) Tumor cells secrete molecules to remodel the tumor microenvironment (TME) after receiving signals through receptors. Epithelial-mesenchymal transition (EMT) occurs in tumor cells, altering the extracellular matrix (ECM) composition and promoting matrix degradation and the formation of new capillaries. These events create a TME conducive to tumor growth and invasion. (2) Tumor cells enter the circulatory system through the close connection of vascular and lymphatic endothelial cells to become circulating tumor cells (CTCs). A few CTCs can intravasate into the capillary network for metastasis with the help of platelets [41], neutrophils[42, 43], endothelial cells, etc. (3) Tumor cells increase endothelial permeability by making endothelial cells contract or destroying their integrity, allowing the tumor cells to extravasate from the circulatory system into organs to form metastases[44]. (4) The proliferative ability of cancer stem cells is restored, and a new premetastatic niche (PMN) is formed in the metastatic target organ, ultimately resulting in tumor metastasis. Exosomes play an indispensable role in this process (Fig. 2).

First, exosomes cause changes in the TME of the primary tumor. PC cells regulate peripheral stromal cells and peripheral tumor cells through exosomes. Researchers found that long intergenic noncoding RNA ROR (linc-ROR) in PC-derived exosomes activates adipocytes in the TME and causes adipocytes to dedifferentiate into preadipocyte/fibroblast-like cells by releasing the cytokine interleukin-1β(IL-1β), which in turn maintains the growth and metastasis of PC cells via the hypoxia inducible factor(HIF) 1α- Zincfinger Ebox Binding Homeobox 1(ZEB1) axis[45]. In addition, under hypoxic conditions, microRNA-301 A-3p in PC cell-derived exosomes downregulates phosphatase and tensin homolog (PTEN) expression in macrophages and activates the PI3Kγ signalling pathway to induce M2 polarization of macrophages, thereby promoting invasion and metastasis in PC[46]. Furthermore, PC cells can downregulate the expression of microRNA-let-7 in pancreatic stellate cells (PSCs) by secreting lin28B via exosomes, thus promoting the expression of high mobility group AT-hook2(HMGA2) and platelet-derived growth factor B(PDGFB). Eventually, PDGDB binds to platelet-derived growth factor receptor (PDGFR) on the surface of PSCs, which leads to recruitment of PSCs to the tumor[47]. Even highly invasive PC cells transfer microRNA-125b-5p into weakly invasive PC cells through exosomes and induce inhibition of STARD13, thereby promoting their invasiveness and metastasis[48]. Simultaneously, the cellular components of the TME also promote the malignant behaviours of PC through exosomes. Hypoxia upregulates the expression of microRNA-4465 and microRNA-616-3p in PSC-derived exosomes, which can be taken up by PC cells and inhibit the PTEN/ AKT signalling pathway, thereby promoting the invasiveness and metastasis of PC cells[49]. MicroRNA-5703 transferred in PSC-derived exosomes can also target chemokine-like factor (CKLF) like MARVEL transmembrane domain containing 4 (CMTM4) expression in PC cells, downregulating its expression and subsequently upregulating p21 (RAC1) activated kinase 4(PAK4) expression, in turn activating the PI3K/AKT signalling pathway to promote cell proliferation [50].

Exosomes promote the intravasation of PC cells into the circulatory system. Li J and his team found that during their entry into the circulatory system, PC cells transfer circRNA IARS (CIRC-IARS) into human microvascular vascular endothelial cells (HUVECs) through exosomes, and CIRC-IARS specifically sponges microRNA-122 in HUVECs, alleviating its inhibition of its target gene Ras homolog gene family, member A(RhoA). This upregulates the expression of RhoA, which further reduces Zonula occludens-1(ZO-1) expression, thereby increasing F-actin expression and endothelial monolayer permeability. Finally, these events enhance vascular invasion and metastasis in PC[51].

The metastatic target organs are altered by exosomes. Costa-silva B’s team found that PC cells transfer macrophage migration inhibitory factor (MIF) into Kupffer cells in the liver through secreted exosomes, inducing transforming growth factor beta (TGF-β) signalling pathway and leading to hepatic stellate cells (HstCs) activation and ECM remodelling. In turn, Fibronectin (FN) accumulation promotes an influx of bone marrow-derived macrophages into the liver, which promotes the formation of a PMN in the liver[52]. Li Z’s group also found that PC cells transfer the long noncoding RNA (lncRNA) Sox2ot to recipient cells through exosomes and that Sox2ot then competitively binds to the microRNA-200 family in recipient cells to regulate Sox2 expression, thereby inducing EMT and stemness properties and ultimately increasing invasion and metastasis in PC[53]. In general, exosomes are involved in many processes of invasion and metastasis in PC.

### Exosomes promote the development of chemoresistance

**Fig. 3:**
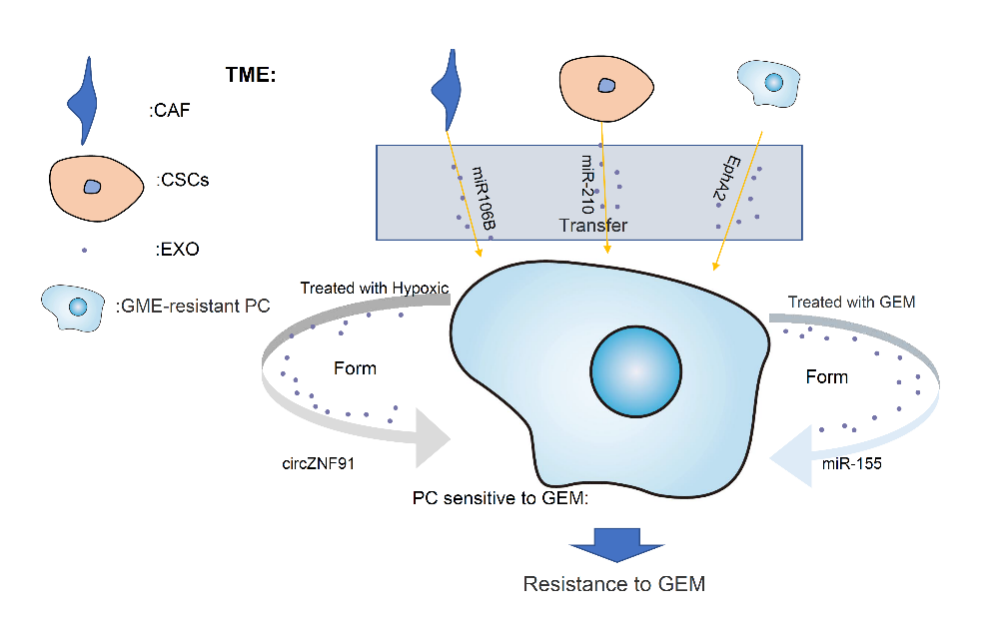
The development of GEM resistant in PC

Surgical resection combined with adjuvant systemic chemotherapy currently provides the only chance for long-term survival in patients with PC [54]. However, the vast majority (80–85%) of patients lose the opportunity for surgery because their PC is already at an advanced stage at diagnosis[55]. The current first-line chemotherapy protocol for PC is gemcitabine (GEM) plus paclitaxel-albumin and FOLFIRINOX (5-fluorouracil, leucovorin, irinotecan, and oxaliplatin), which are the mainstays of treatment for patients with advanced PC[4, 54]. However, in PC, the unique pathobiological limitations caused by the ECM and the extreme lack of tumor-associated angiogenesis hinder the penetration of chemotherapeutic drugs [56–58]. In addition, chemoresistance, especially the problem of GEM resistance [57, 59] seriously reduces the benefits of these approaches. In PC, cancer cells and other cells in the TME promote the development of chemoresistance through exosomal regulation of RNAs, proteins, and related signalling pathways[60] (Fig. 3).

In the TME of PC, there are many cells that are inherently resistant to GEM, including cancer-associated fibroblasts (CAF) [58, 61] and Cancer Stem cells (CSCs)[61], which can transfer chemoresistance through exosomes. Patel GK’s team found that CAF-derived exosomes in PC are rich in microRNA106B and can transfer microRNA106B into PC cells through exosomes, thereby reducing the tumor protein p53 inducible nuclear protein 1 (TP53INP1) protein level in PC cells and inducing GEM resistance in tumor cells[62]. In addition, Yang Z’s group found that CSCs can deliver microRNA-210 into GEM-sensitive PC cells through exosomes, activating the mTOR signalling pathway and enabling GEM-sensitive tumor cells to develop resistance to GEM[61]. Moreover, Fan J’s team confirmed that GEM-resistant PC cells can transfer Ephrin type-A receptor 2(EphA2) into GEM-sensitive PC cells through exosomes, leading to the transfer of chemoresistance[63].

In addition to the abovementioned acquisition of GME resistance through the transfer of chemoresistance via exosomes, PCs can also acquire chemoresistance through the uptake of exosomes. For example, long-term exposure to GEM can upregulate the expression of microRNA-155 in PC cells. In addition to promoting the anti-apoptotic activity and increasing the chemoresistance ability of tumor cells by acting on target genes, microRNA-155 can be transferred to other cancer cells via exosomes, endowing the recipient cells with chemoresistance[64]. Furthermore, during GEM treatment, PC cells suppress the effects of GEM by downregulating the expression of the key gemcitabine-metabolizing enzyme deoxycytidine kinase (DCK) via exosomal microRNA-155, simultaneously increasing the expression of catalase (CAT) and superoxide dismutase 2 (SOD2), which reduces reactive oxygen species (ROS) levels to induces chemotherapeutic resistance and inhibit cell death [65]. Moreover, hypoxia can induce PC cells to release exosomes rich in CircZNF91. CircZNF91 upregulates Sirtuin1 (SIRT1) expression by competitively binding to microRNA-23b-3p in normoxic PC cells and enhances the deacetylation-dependent stability of the HIF-1α protein. This promotes glycolysis and chemoresistance in PC cells[66]. In general, exosomes play a role in promoting the development of chemoresistance in PC.

### Exosomes promote the formation of an immunosuppressive environment in PC

**Fig. 4:**
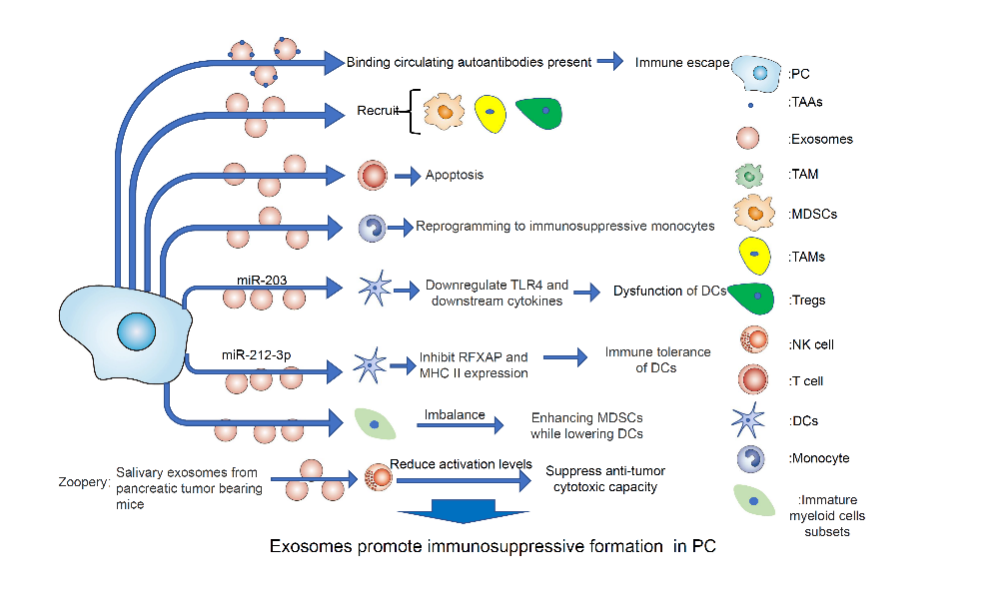
Exosomes promote the formation of an immunosuppressive environment in PC

The immune system, which includes lymphoid organs, cells, cytokines, etc., is an essential tool in host defence [67]. The immune system recognizes and eliminates threats to maintain homeostasis and provide protection against exogenous and endogenous diseases, including infections and tumors[68, 69]. However, PC patients’ immune systems are altered by exosomes[70] (Fig. 4).

First, exosomes are inherently immunogenic. Their surfaces are rich in tumor-associated antigens (TAAs), such as annexin A1 (ANXA1), keratin 10 (KRT10), keratin 16 (KRT16), and tubulin beta class I (TUBB), which further deplete antibodies and associated immune cells by binding to circulating autoantibodies. In turn, complement-dependent cytotoxicity and potential antibody-dependent cell-mediated cytotoxicity are suppressed[71].

Second, the TME is regulated by exosomes [72]. In PC, tumor cells remodel the TME through exosomal cargos, including proteins, lncRNAs, messenger RNAs (mRNAs), and microRNAs (MiRNAs), to recruit immunosuppressive inflammatory cells. For example, tumor-associated macrophages (TAMs), bone marrow-derived suppressor cells (MDSCs), and regulatory T cells (Tregs) contribute to the formation of an immunosuppressive environment [73]. In addition, PC-derived exosomes regulate normal immune cells, including T lymphocytes, natural killer cells (NKs), and dendritic cells (DCs), to change their original functions[74]. PC-derived exosomes reprogram normal monocytes into immunosuppressive monocytes by downregulating human leukocyte antigen-DR(HLA-DR), altering STAT3 signalling pathway, and inducing arginase expression and reactive oxygen species (ROS) production[70]. Exosomes are also taken up by T lymphocytes and activate the phosphorylation of P38 mitogen-activated protein kinase (MAPK) in T lymphocytes, which induces endoplasmic reticulum (ER) stress activation followed by activation of the PERK-eIF2 α-ATF4-CHOP signalling axis, in turn inducing apoptosis in T lymphocytes[75]. In addition, exosomes can regulate the activity of DCs, NKs, and other cells. Researcher found that PC cells can inhibit the expression of regulatory factor X-associated protein (RFXAP) by exosomal transfer of microRNA-212-3p into DCs, thereby reducing the expression of MHC II molecules and inducing DC-related immunotolerance[72, 76]. PC-derived exosomes also inhibit the expression of Toll-like receptor 4 (TLR4) and tumor necrosis factor-alpha (TNF-α) on DCs by transferring microRNA-203. This leads to DC dysfunction[74]. Furthermore, exosomes derived from PC can disbalance immature bone marrow cell subsets, including increase the proportion of MDSCs and decrease the proportion of DCs by altering intracellular calcium flux in the presence of SMAD4 inhibition[77]. Moreover, the ability of exosomes secreted from other organs of individuals with PC (e.g., saliva) to reduce NKs activation has also been shown in animal studies[78]. In general, exosomes alter the ability of immune cells to fight tumors so that PC cells acquire the power of immunosuppression.

## Clinical applications of exosomes in PC

As the understanding of the contents, structure, and characteristics of exosomes and the pathological mechanism of PC has increased, we have confirmed that exosomes have broad prospects in clinical application in PC. At present, two problems in the clinical treatment of PC that urgently need to be overcome are finding a suitable biomarker for PC and developing a novel therapy that can improve the therapeutic effect in PC patients.

### Exosomes can be used as biomarkers for PC

The poor prognosis of PC is mainly due to delayed diagnosis, early metastasis and frequent recurrence[2, 3]. More than 2000 biomarkers for these events have been studied in PC [79]. However, Carbohydrate antigen 19 − 9(CA19-9) is the only Food and Drug Administration (FDA)-approved biomarker for PC[80]. Still, CA19-9 has some disadvantages, such as its insufficient specificity and sensitivity[79], especially its poor ability to distinguish benign pancreatic diseases from PC. Therefore, it is urgent to find a more sensitive and more specific biomarker that can best detect recurrence and treatment effectiveness. Researchers are becoming increasingly convinced that exosomes are excellent biomarkers for PC because of their properties (Table 1).

**Table 1:** Exosomes can be used as biomarkers

Type	Name	Sensitivity	Specificity	AUC	Effect	References
Early detection	EXO-GPC1	100%	100%	1	Early detection, Disease stage and Prognosis prediction	[81]
	EXO-WASF1	NT	NT	0.943	Early detection	[82]
	EXO-ARF6	NT	NT	0.94	Early detection	[82]
	EXO-c-Met	70%	85%	0.779	Early detection	[83]
	EXO-c-Met + CA19-9	72%	90%	>0.779	Early detection	[83]
	EXO-EphA2	83%	94%	0.94	Early detection, Judge benign and malignant	[85]
	EXO-EphA2 + CA19-9 + CA242	92.50%	98.50%	0.98	Early detection, Judge benign and malignant	[85]
	CA19-9	87.40%	97.20%	0.95	Standard biomarker	[85]
	EXO- HIST2H2AA3/ LUZP6/ HLA-DRA	NT	NT	0.8558	Early detection, Judge benign and malignant	[84]
Prognostic assessment	EXO-integrins	NT	NT	NT	Predicting organotropic metastasis	[86]
	EXO-MicroRNA-451a in Portal vein blood	72.70%	77.30%	NT	High-risk of recurrence and poor survival following surgery	[87]
	EXO-MicroRNA-21 in Portal vein blood	72.70%	72.70%	NT	High-risk of recurrence and poor survival following surgery	[87]
	EXO-MicroRNA-4525 in Portal vein blood	81.80%	86.40%	NT	High-risk of recurrence and poor survival following surgery	[87]
	EXO-MIF	NT	NT	NT	Predicting liver metastasis	[52]
Not tested = NT						

Exosomes as biomarkers can be divided into two major parts: diagnostic markers and prognostic markers. as diagnostic markers: GPC1, WASF1, ARF6, C-MET, C-Met + CA19-9, EphA2, EphA2 + CA19-9 + CA242, CA19-9 and HIST2H2AA3/LUZP6/HLA-DRA. As prognostic markers: integrins, MicrorNA-451 A in Portal vein Blood, MicroRNA-21 in Portal vein Blood, MicroRNA-4525 in Portal vein blood, MIF

#### Exosomes are markers for early diagnosis of PC

Prof. Melo SA’s team used exosomal surface glypican-1 (GPC-1) as a biomarker for early diagnosis of PC. The diagnostic sensitivity, specificity, and positive and negative predictive values were all 100%, and the area under curve (AUC) was 1. Although the included data were not sufficient, these data demonstrate the potential of exosomes as biomarkers[81]. Good sensitivity and specificity and high AUC values have also been obtained in studies using WASP family member 2(WASF2), ADP ribosylation factor 6(ARF6), proto-oncogene mesenchymal-epithelial transition factor(C-Met), and other exosomal proteins as biomarkers for PC [82, 83]. In addition, Yixing Wu’s team used gene expression profiles from public databases, and found that the expression levels of histone cluster 2 H2A family member A3(HIST2H2AA3), leucine zipper protein 6 (LUZP6) and major histocompatibility complex, class II, DR alpha (HLA-DRA) in the exosome displayed high value in distinguishing PC from both healthy people (AUC = 0.8558) and chronic pancreatitis (AUC = 0.815)[84].

Combinations of exosomes and other biomarkers can further improve the accuracy of diagnosis. One of the major disadvantages of CA19-9 is its poor ability to distinguish benign pancreatic diseases from PC. The study of Wei Q’s team using exosome-EphA2 + CA19-9 + carbohydrate antigen 242(CA242) as an early diagnostic marker for PC showed that the combination of exosome-EphA2 + CA19-9 + CA242 can improve not only the ability for early diagnosis of PC but also the ability to distinguish between PC and benign pancreatic diseases[85].

#### Exosomes are prognostic markers for PC

In addition to the ability for early diagnosis, exosomes also have a good ability for prognostic assessment. Hoshino Ad’s team found that the levels of integrins in exosomes can be used to predict tumor metastasis to different organs[86]. Furthermore, high levels of macrophage migration inhibitor (MIF) in exosomes predicts liver metastasis in PC[52]. Moreover, Kawamura S’s team found that the levels of microRNA-4525, microRNA-451 A, and microRNA-21 in portal vein-derived exosomes predicted the risk of recurrence and survival after surgery[87].

### Exosomes play an important role in the development of novel therapies for PC

**Fig. 5:**
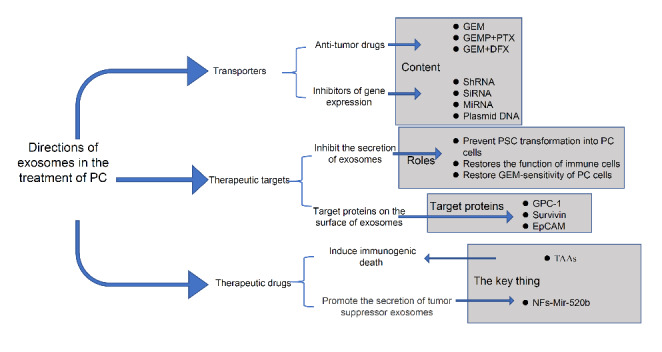
Directions in exosome therapy for PC:

Exosomes are nanoscale vesicles secreted by cells that have the advantages of high biocompatibility, fast ECM traversal[14], long retention time[88], and low toxicity. Combined with the characteristics of exosomes, its application in the treatment of PC mainly has the following directions[60] (Fig. 5): (1) Exosomes can be used as transporters; (2) Exosomes can be used as therapeutic targets; (3) Exosomes can be used as therapeutic drugs.

#### Exosomes can be used as transporters

The application of exosomes as transporters can be divided into two main approaches. The first approach is the delivery of antitumor drugs, including chemotherapeutic drugs, through exosomes. The TME of PC has a dense connective tissue hyperplasia response[77] and an unusually abundant ECM. This unique microenvironment seriously hinders the entry of chemotherapeutic drugs and affects the efficacy of chemotherapy [57]. Li YJ’s team compared the antitumor effects of GEM alone and GEM loaded into exosomes and eventually found that the treatment regimen of GEM-loaded exosomes showed superior therapeutic effects and prolonged survival with minimal damage to normal tissues[89]. Zhou Y’s team developed a combined gemcitabine monophosphate (GEMP) and paclitaxel (PTX) delivery platform using bone marrow mesenchymal stem cell (BM-MSC)-derived exosomes as vectors to overcome chemoresistance and pathological barriers in PC treatment[57]. Zhao Y’s team also coloaded GEM with Deferasirox (DFX) into M1 macrophage-derived exosomes (M1 Exos) as drug carriers. This regimen significantly improved the therapeutic effect in PC, especially GEM-resistant PC, by inhibiting cancer cell proliferation, adhesion, migration, and chemoresistance[90].

The second approach is to use exosomes to transport inhibitors of gene expression, such as small interfering RNAs (SiRNAs), to interfere with the expression of PC-related genes and thus play a role in the cure of PC. Currently, genetic mutations are thought to play an important role in the development of PC. Among commonly mutated genes, KRAS, CDKN2A, TP53, and SMAD4 are the driver genes in PC, and mutations in KRAS and CDKN2A are early events in PC initiation[3, 4]. Kamerkar S used exosomes as transport vehicles to deliver KRASG12D-targeted SiRNA or short hairpin RNA (ShRNA) in various PC mouse models. This strategy significantly reduced KRASG12D mRNA levels and phosphorylated ERK protein levels in the tested PC cell line, which inhibited tumor metastasis and prolonged survival[91]. In both subcutaneous xenograft and in situ models, McAndrews KM’s team inhibited PC proliferation and tumor growth by targeting the KRASG12D oncogenic mutation with exosomes loaded with CRISPR/Cas9 plasmid DNA[92]. In addition, targeting BCL2 apoptosis regulator (BCL2) with exo-microRNA-34a significantly enhanced the expression of proapoptotic proteins such as BCL2 associated X, apoptosis regulator (Bax) and P53, which in turn inhibited tumor growth by promoting apoptosis[93]. In addition, exosomes were found to inhibit tumor proliferation and invasion by regulating Smad3 in PC by transporting microRNA-145-5p [94].

#### Exosomes can be therapeutic targets

There are two main approaches to the use of exosomes as therapeutic targets. The first approach is to inhibit the secretion of exosomes, and the second is to target proteins on the surface of exosomes. As mentioned earlier, exosomes promote the malignant characteristics of PC, and we can inhibit the secretion of exosomes to achieve antitumor goals. For example, inhibition of related exosome secretion was found to prevent PSC transformation into PC cells[47], restore NKs activation levels[78], ameliorate GEM resistance induced by exosomal cargos such as microRNA-155[64], and so on. As stated above, the second approach is to target proteins on the surface of exosomes for antitumor purposes. Some surface proteins are significantly associated with poor prognosis in cancers; for example, Aikawa T’s team demonstrated that downregulation of the surface glycoprotein GPC1 inhibits tumor angiogenesis and metastasis[95]. In addition, Chang WH’s group found that Survivin expression on the exosome surface can effectively inhibit the malignant characteristics of PC [96]. In addition, drugs such as edrecolomab and catumaxomab that target epithelial cell adhesion molecule (EpCAM), a surface protein found to be specifically expressed on exosomes isolated from PC patients, have been used in antitumor studies[97].

#### Exosomes can be used as therapeutic drugs

Exosomes can be used as therapeutic drugs because of two characteristics. The first is that exosomes themselves have immunogenicity, and the surface of exosomes contains many TAAs[71], which can stimulate an antitumor immune response and thus induce immunogenic cell death (ICD) in tumor cells. Jang Y’s group discovered a novel strategy for treating PC using tumor-derived exosomes to stimulate antitumor immune responses[98]. In addition, Zhou W’s group developed a drug delivery system based on BM-MSC-derived exosomes. This strategy improves DC maturation, reverses immunosuppression, increases the infiltration of antitumor cytotoxic T lymphocyte, and induces effective innate and adaptive immune responses against PC[56]. Second, researchers have found that some exosome cargos can inhibit the malignant behaviours of PC cells. Shi H’s group found that microRNA-520b (miR-520b) in exosomes produced by normal fibroblasts (NFs) inhibits PC cell proliferation, migration, and invasion by downregulating ZNF367[99].

### Exosomes’ benefits and drawbacks in clinical application of PC

At present, the therapeutic effect of PC has been improved since the strategy developed from surgery to multidisciplinary treatment. However, the overall survival is still frustrating with the low rate of early diagnosis. Finding suitable biomarkers is critical to improve the early diagnosis of PC. Exosome research points to a new direction in searching for PC biomarkers[84, 100]. Exosomes as biomarkers have the following advantages: First, exosomes are secreted in a large amount by PC tumors and presented in multiple body fluids, which may make PC detection more sensitive. Second, exosomes can be real-time as the contents and quantity vary with the tumor. Thus, exosomes may be more predictive and prospective. Third, the composition of exosomes is highly homologous to secretory cells, which may promote higher specificity of exosome-based detection[101]. These features are essential for early diagnosis, disease progression detection, and recurrence detection of PC, which are also guidelines for research on exosomes as biomarkers.

Various roles of exosomes are being discovered, and the regulatory effects of exosomes on malignant characteristics such as invasion, metastasis, immunosuppression, and chemoresistance in cancers, including PC, have been proven. Exosomes revealed many advantages, such as high biocompatibility, fast ECM traversal[14], long retention time[88], and low toxicity. Drug carriers, therapeutic targets, and therapeutic drugs developed based on exosomes against PC have shown encouraging outcomes in cellular trials. Meanwhile, several clinical trials are underway (NCT03608631/ NCT03410030).

However, exosome research does encounter some difficulties. Although the mainstream believes that the biogenesis of exosomes is endogenous pathways, some scholars have proposed that exosomes are secreted through budding[102], which undoubtedly has a significant impact on the basic theory of exosomes. Moreover, isolating and extracting exosomes efficiently is also an urgent problem to be solved. Microfluidic technology and a combination of multiple extraction methods provide a novel way to solve this problem. In addition, applying these exosome research achievements to clinical patients still need a long way to go due to the epigenetic, unusual TME and biological behavior of PC.

## Conclusions

This review systematically describes the biogenesis process, extraction methods, and detection strategies of exosomes, provides a complete explanation of the roles and mechanisms of exosomes in the malignant characteristics of PC, and describes the clinical application of exosomes in PC based on the above observations. In particular, the application of exosomes as biomarkers for PC and the general directions for the use of exosomes in the treatment of PC are described, providing a good guide for the development of new diagnostic tools and treatment plans for PC by utilizing the characteristics of exosomes. The research and utilization of exosomes also face some problems, such as the great technical difficulty, preliminary stage of clinical trials, insufficiency of mechanistic research, etc. These are also problems to be solved in our subsequent work. In addition, applying the results of exosome research to the clinical treatment of PC and better serving patients requires further research and exploration.

### Electronic supplementary material

Below is the link to the electronic supplementary material.

Supplementary Material 1

Supplementary Material 2
