# Correction: Functions and clinical applications of exosomes in Pancreatic cancer

**DOI:** 10.1007/s11033-023-08906-3

**Published:** 2023-11-10

**Authors:** Zhichen Jiang, Huiju Wang, Yiping Mou, Li Li, Weiwei Jin

**Affiliations:** 1 Department of General Surgery, Devision of Gastroenterology and Pancreas, Zhejiang Provincial People’s Hospital, Affiliated People’s Hospital, Hangzhou Medical College, 310014 Hangzhou, Zhejiang China; 2 The Second School of Clinical Medicine, Zhejiang Chinese Medical University, 310053 Hangzhou, Zhejiang China; 3 Key Laboratory of Gastroenterology of Zhejiang Province, 310014 Hangzhou, Zhejiang China; 4 Cancer Center, Zhejiang Provincial People’s Hospital, Affiliated People’s Hospital, Hangzhou Medical College, 310014 Hangzhou, Zhejiang China

**Correction: Molecular Biology Reports (2022) 49:11037–11,048**

10.1007/s11033-022-07765-8.

In this article, the statement in the Funding information section was incorrectly given as ‘This study was supported by grants from the Zheji-ang Provincial Basic Technology Foundation of China (Grant No.GC20H160003)’.

The correct statement should read as ‘This study was supported by grants from the Zhejiang Provincial Basic Technology Foundation of China (Grant No.LGC20H160003)’. The Acknowledgements section is removed.

The original article has been corrected.

