# Supplementary material for: Functions and clinical applications of exosomes in pancreatic cancer
Source: Mol Biol Rep. 2022 Sep 12;49(11):11037–48. doi: 10.1007/s11033-022-07765-8 (PMC9618535; doi:10.1007/s11033-022-07765-8)
Supplement: Supplementary file 1 — Supplementary Material 1 [file 11033_2022_7765_MOESM1_ESM.pdf]

Editing Certificate

This document certifies that the manuscript

Recent Advances of exosome in pancreatic cancer

prepared by the authors

Zhichen Jiang,Weiwei Jin,Huiju wang,Yiping Mou,Li Li

was edited for proper English language, grammar, punctuation, spelling, and overall style by one or more of the highly qualified native English speaking editors at AJE.

This certificate was issued on **March 11, 2022** and may be verified on the AJE website using the verification code **A228-5021-0D8C-44BF-0CFD**.

Neither the research content nor the authors' intentions were altered in any way during the editing process. Documents receiving this certification should be English-ready for publication; however, the author has the ability to accept or reject our suggestions and changes. To verify the final AJE edited version, please visit our verification page at aje.com/certificate. If you have any questions or concerns about this edited document, please contact AJE at support@aje.com.

AJE provides a range of editing, translation, and manuscript services for researchers and publishers around the world. For more information about our company, services, and partner discounts, please visit aje.com.
