# Supplementary material for: Functions and clinical applications of exosomes in pancreatic cancer
Source: Mol Biol Rep. 2022 Sep 12;49(11):11037–48. doi: 10.1007/s11033-022-07765-8 (PMC9618535; doi:10.1007/s11033-022-07765-8)
Supplement: Supplementary file 2 — Supplementary Material 2 [file 11033_2022_7765_MOESM2_ESM.docx]

| **Supplemental table 1: Extraction methods and characteristics of exosomes** | | | |
| --- | --- | --- | --- |
| Delegate Methods | Theory | Advantage | Disadvantage |
|  |  |  |  |
| Ultracentrifugation, Density gradient centrifugation | Density difference | Straightforward | Low yield, Time-consuming and Damage exosomes |
| Sequential filtration, Ultrafiltration, and SEC | Particle size difference | Efficient and Ease of implementation | Damage exosomes, Vesicles blocking filter membrane |
| Immunocaptured Technique/Heparin Magbeads | Antigen-antibody reaction | High specificity | Expensive and High sample requirements |
|  |  |  |  |
| Polyethylene glycol(PEG) precipitate method，Protamine aggregation method | Precipitation | Convenient operation | Low purity and specificity |
|  |  |  |  |
| [Microfluidic chip analysis](javascript:;) | Microfluidic technology | High resolution and sensitivity, Fast determination time | Technical difficulty |

| **Supplemental table 2: Types of extracellular vesicles** | | | | | |
| --- | --- | --- | --- | --- | --- |
| Name | Size | Origin | Marker | Biogenesis | Density |
| Exosome | 40-150nm | endosomes | CD81、CD63、CD9 | The endocytic pathway | 1.13-1.19g/ml |
| Microvesicle | 100-1000nm | plasma membrane | [CD40、integrins](javascript:;) | bud | 1.16-1.19g/ml |
| apoptotic body | 1000-5000nm | plasma membrane、endoplasmic reticulum | phosphatidylserine、genmic DNA | bud、byzelosls、 | 1.16-1.28g/ml |
| migrasome | 500-3000nm | plasma membrane | tetraspanin 4 (TSPAN4)、integrins | migracytosis | NS |
|  |  |  |  | Not sure=NS | |
